# Dose of nafamostat mesylate during continuous kidney replacement therapy in critically ill patients: a two-centre observational study

**DOI:** 10.1186/s12882-024-03506-0

**Published:** 2024-02-26

**Authors:** Shinya Kameda, Akinori Maeda, Shun Maeda, Yutaro Inoue, Kazunari Takahashi, Akira Kageyama, Kent Doi, Tomoko Fujii

**Affiliations:** 1 Intensive Care Unit, The Jikei University Hospital, 3-19-18 Nishi-Shimbashi, Minato-ku, 105-8471 Tokyo, Japan; 2 Department of Emergency and Critical Care Medicine, The University of Tokyo, Tokyo, Japan; 3 Department of Pharmacy, The Jikei University Hospital, Tokyo, Japan

**Keywords:** Acute kidney injury, Continuous kidney replacement therapy, Nafamostat mesylate, Filter life, Anticoagulant, Dose

## Abstract

**Background:**

Nafamostat mesylate is an anticoagulant used for critically ill patients during continuous kidney replacement therapy (CKRT), characterised by its short half-life. However, its optimal dosage remains unclear. This study aimed to explore the optimal dosage of nafamostat mesylate during CKRT.

**Methods:**

We conducted a two-centre observational study. We screened all critically ill adult patients who required CKRT in the intensive care unit (ICU) from September 2013 to August 2021; we included patients aged ≥ 18 years who received nafamostat mesylate during CKRT. The primary outcome was filter life, defined as the time from CKRT initiation to the end of the first filter use due to filter clotting. The secondary outcomes included safety and other clinical outcomes. The survival analysis of filter patency by the nafamostat mesylate dosage adjusted for bleeding risk and haemofiltration was performed using a Cox proportional hazards model.

**Results:**

We included 269 patients. The mean dose of nafamostat mesylate was 15.8 mg/hr (Standard deviation (SD), 8.8; range, 5.0 to 30.0), and the median filter life was 18.3 h (Interquartile range (IQR), 9.28 to 36.7). The filter survival analysis showed no significant association between the filter life and nafamostat mesylate dosage (hazard ratio 1.12; 95 CI 0.74–1.69, *p* = 0.60) after adjustment for bleeding risk and addition of haemofiltration to haemodialysis.

**Conclusions:**

We observed no dose-response relationship between the dose of nafamostat mesylate (range: 5 to 30 mg/h) and the filter life during CKRT in critically ill patients. The optimal dose to prevent filter clotting safely needs further study in randomised controlled trials.

**Trial registration:**

Not applicable.

**Supplementary Information:**

The online version contains supplementary material available at 10.1186/s12882-024-03506-0.

## Background

Continuous kidney replacement therapy (CKRT) is a frequent intervention in the intensive care units (ICU) [1]. CKRT is performed assuming a filter life of > 24 h, with anticoagulants commonly used. However, CKRT is frequently interrupted for circuit replacement due to intra-circuit clotting, leading to blood loss, increased staff workload, costs, and decreased therapeutic efficacy [2]. To avoid such treatment interruption, an adequate dose of anticoagulant should be used; however, it inherits the risk of bleeding adverse events.

Recent clinical guidelines recommend using regional citrate anticoagulation or heparin according to the bleeding risks of patients [3]. However, citrate-based dialysis is not available everywhere, and some patients must avoid heparin due to allergy or bleeding risks. Therefore, it is imperative to have alternatives for the anticoagulation strategies; in this regard, nafamostat mesylate is a potential candidate, given its favourable features.

Nafamostat mesylate is a serine protease inhibitor with a short half-life of 8 min [4] and is expected to take effect locally within the circuit [5, 6]. Nafamostat mesylate is known to inhibit the activity of a range of proteases, including thrombin, in the complement system, and factors VIIa, Xa, and XIIa in the coagulation system (eFigure 1) [7]. In some countries, nafamostat mesylate is used as an anticoagulant during CKRT, primarily because citrate-containing dialysate is not available and nafamostat mesylate has historically been based on empirical practice [8].Some studies reported using nafamostat mesylate at 20 mg/h during CKRT [4, 9, 10]; however, the reported doses vary across previous studies. Moreover, the optimal dosage of nafamostat mesylate has yet to be investigated, and the evidence base of the doses used in the previous studies is unclear.

This study aimed to investigate the relationship between nafamostat mesylate dosage and filter life and explore the optimal dosage of nafamostat mesylate during CKRT.

## Methods

We conducted an observational study in two ICUs of university-affiliated hospitals. We screened critically ill adult patients in the ICU who underwent CKRT from September 2013 to August 2021either for acute kidney injury (AKI) or chronic dialysis. The inclusion criteria were age of 18 years or older and the administration of nafamostat mesylate to the circuit of CKRT. We excluded patients who received CKRT for more than 12 h without anticoagulant and were administered unfractionated heparin sodium or sodium citrate during CKRT. We also excluded patients who opted out of the study. The study results are reported according to the STROBE (Strengthening The Reporting of Observational Studies in Epidemiology) checklist (eTable 1) [11].

We collected the following data from electronic medical records and a local ICU database: demographic information (age, sex, height, and weight), past medical history (hypertension, chronic dialysis, ischemic heart disease, heart failure [classified as New York Heart Association Class IV], diabetes requiring insulin,), diagnosis at the ICU admission, emergency or scheduled admission, acute physiology and chronic health evaluation (APACHE) II score, medical or surgical admission, use of nephrotoxic agents, and bleeding risk.

Bleeding risk was defined by any of the following: clinically suspected active bleeding, a haemorrhagic event or surgical operation within 48 h, cerebral haemorrhage within 4 weeks, ischemic stroke within 2 weeks, activated partial thromboplastin time (APTT) > 60 s, prothrombin time-international normalized ratio (PT-INR) > 2.0, or platelet count < 100,000/µL.

We collected data on vital signs at CKRT initiation and 24 h later and the following CKRT specifics: filter type, blood flow rate, dialysate flow rate, filtration flow rate, body fluid removal rate, date and time of filter usage, nafamostat mesylate dosage at CKRT start, and the reason for ending filter use. Artrial blood gas analysis and blood test data at CKRT start and 24 h later were also collected. To assess the risk of bleeding adverse events, transfusion volumes within 48 h after CKRT initiation were collected. The dosing of nafamostat mesylate was determined empirically, relying on the discretion and clinical judgment of individual hospitals, physicians, or technicians involved in usual clinical practice.

The primary outcome was filter life. Filter life was defined as the duration from CKRT initiation to the end of the first filter use due to clotting. In case the filter use ended for reasons other than clotting, i.e. leaving the ICU for imaging tests, surgical operations, or discharge, the observation of filter life was censored. The secondary outcomes included the ICU length of stay, hospital length of stay, duration of mechanical ventilation, dialysis dependence at discharge for patients who was not on chronic dialysis at ICU admission, ICU mortality, in-hospital mortality, C-reactive protein, and transfusion volumes within 48 h after CKRT initiation.

### Statistical analysis

Continuous data are presented as mean with standard deviation (SD) or median with interquartile range (IQR) where appropriate, and absolute numbers are presented with percentages. The probability of filter patency over time, adjusted for bleeding risk and haemofiltration, was analysed using a Cox proportional hazards model. The analysis categorised the patient population into two groups based on the dosage of nafamostat mesylate: a high-dose group (≥ 20 mg/h) and a low-dose group (< 20 mg/h). The grouping was made at the median value within the range of actual administered doses.

The association between nafamostat mesylate dose and secondary outcomes were also assessed using generalised linear regression analysis or generalised logistic regression analysis. Variables used for the adjustment of confounding factors are reported in the eTable 2.

Given the nature of the observational study to explore the association in clinical settings, sample size calculation was not performed, and all data that were available from electronic health record were used for this study.

All statistical analyses were performed using R ver.4.0.5 (R Foundation for Statistical Computing, Vienna, Austria). *P*-value < 0.05 was considered to be statistically significant.

### Ethics

This study was approved by the Jikei University Ethics Committee (33–368 [10992]). The committee waived the requirement for written informed consent given the retrospective nature of the study. In compliance with the directions of the relevant ethics committee, we posted a notice on the bulletin board of the facility. This notice detailed the nature of the study and explicitly stated that patients had the right to request exclusion from the study (opt out).

## Results

We included 269 critically ill patients who underwent CKRT using nafamostat mesylate as an anticoagulant. Of these, 68.4% were male, and 59.5% had at least one bleeding risk (Table 1). The mean age was 65 years (SD, 15), with a mean APACHE II score of 28 (SD, 7.7). The mean nafamostat mesylate dose was 15.8 mg/h (SD, 8.8). Filters used included polyethylenimine-coated polyacrylonitrile (AN69ST), cellulose triacetate, and polysulfone (Table 1). Laboratory data and the arterial blood gas analysis results at CKRT initiation and after 24 h are presented in Table 2. There was a shift in distribution of APTT with an increase in the mildly prolonged range from 33.1% at baseline to 51.9% at 24 h. pH increased over the 24 h. Table 3 shows details of CKRT delivery, vital signs and catecholamine use at CKRT initiation.

**Table 1:** Baseline characteristics

	Overall (*n* = 269)
Age, years	65 ± 15
Sex	
Male	184 (68.4%)
Female	85 (31.6%)
Height, cm	162.4 ± 10.4
Weight, kg	60.5 ± 17.4
APACHE II score	28 ± 7.7
Admission type	
Medical	211 (78.4%)
Elective surgery	24 (8.9%)
Emergency surgery	34 (12.6%)
Admission route	
Ward	113 (42.0%)
Operation room	52 (19.3%)
Emergency room	69 (25.7%)
Other hospital	35 (13.0%)
Nafamostat mesylate dose, mg/h	15.8 ± 8.8
Chronic medical condition	
Hypertension	163 (60.6%)
Ischemic heart disease	64 (23.8%)
Diabetes mellitus	35 (13.0%)
Maintenance dialysis	87 (32.3%)
Heart failure	11 (4.1%)
Bleeding risk	160 (59.5%)
Creatinine at discharge, mg/dL	3.57 ± 3.42
Nephrotoxic agents	81 (30.1%)
Filter clot	167 (62.1%)
Filter type	
AN69ST	74 (27.5%)
CTA	19 (7.1%)
PS	176 (65.4%)

Values are presented as means with standard deviations, otherwise specified

APACHE denotes acute physiology and chronic health evaluation; AN69ST, polyethylenimine-coated polyacrylonitrile; CTA, cellulose triacetate; PS, polysulfone. Nephrotoxic agents included Non-Steroidal Anti-Inflammatory Drugs (NSAIDs), amphotericin B, aminoglycosides, cyclosporine, tacrolimus, or contrast media which patients were exposed to within 48 h prior to CKRT.

**Table 2:** Laboratory data and blood gas sampling data at the start of CKRT and at 24 h

	Baseline	24 h
Albumin, g/dL	2.6 ± 0.7	2.5 ± 0.6
Haemoglobin, g/dL	9.8 ± 2.4	9.2 ± 1.8
Platelet, ×10^3^/µl	142 ± 113	112 ± 88
PT-INR		
< 1.5	205 (76.2%)	206 (77.2%)
> 1.5	64 (23.8%)	61 (22.8%)
APTT sec		
< 40	170 (64.6%)	111 (41.4%)
40–80	87 (33.1%)	139 (51.9%)
> 80	6 (2.3%)	18 (6.7%)
Serum creatinine, mg/dL	4.58 ± 3.65	3.33 ± 2.67
Calcium, mg/dL	7.9 ± 1.1	8.3 ± 1.0
Magnesium, mg/dL	2.3 ± 0.6	2.2 ± 0.6
Inorganic phosphate, mg/dL	5.4 ± 2.6	4.1 ± 1.8
White blood cell,×10^3^/µl		
< 3	13 (4.9%)	18 (6.8%)
3–9	81 (30.5%)	86 (32.3%)
> 9	172 (64.7%)	162 (60.9%)
C-reactive protein, mg/dL	9.3 ± 10.3	10.7 ± 10.8
FiO2	0.37 ± 0.21	0.34 ± 0.14
pH	7.33 ± 0.11	7.37 ± 0.39
PaO2, mmHg	111.0 ± 63	93.8 ± 34
PaCO2, mmHg	35.3 ± 10.4	37.2 ± 9.1
Bicarbonate, mmol/L	18.5 ± 5.6	22.0 ± 4.3
Sodium, mmol/L	137.0 ± 7.3	137.3 ± 4.6
Potassium, mmol/L	4.8 ± 1.0	4.3 ± 0.7
Chloride, mmol/L	106.6 ± 7.6	106.8 ± 4.5
Lactate, mmol/L	3.7 ± 5.9	2.8 ± 3.6

Values are presented as means with standard deviations, otherwise specified

CKRT denotes continuous kidney replacement therapy; PT-INR, prothrombin time international normalised ratio; APTT, activated partial thromboplastin time; FiO2, fraction of inspiratory oxygen; PaO2, partial pressure of arterial oxygen; PaCO2, partial pressure of arterial carbon dioxide

**Table 3:** CKRT settings, vital signs, interventions

Variables	*n* = 269
Blood flow rate: QB, ml/min	106.0 ± 12.9
Continuous haemodiafiltration (CHDF)	115 / 269 (42.8%)
Dialysate flow rate: QD, mL/kg/h	22.3 ± 13.4
Filtration rate: QF, ml/kg/h	9.7 ± 4.3
Continuous haemodialysis (CHD)	154 /269 (57.2%)
Dialysate flow rate: QD, mL/kg/h	18.3 ± 15.8
Fluid removal rate, ml/h	49.0 ± 112.4
Mean arterial pressure at the start of CKRT, mmHg	81.1 ± 19.6
Heart rate at the start of CKRT, bpm	94.3 ± 22.3
Respiratory rate at the start of CKRT, /min	21.1 ± 6.7
Intervention	
Noradrenaline at the start of CKRT	132 (49.1%)
Vasopressin at the start of CKRT	45 (16.7%)

Values are presented as means with standard deviations, otherwise specified

CKRT denotes continuous kidney replacement therapy

**Fig. 1:**
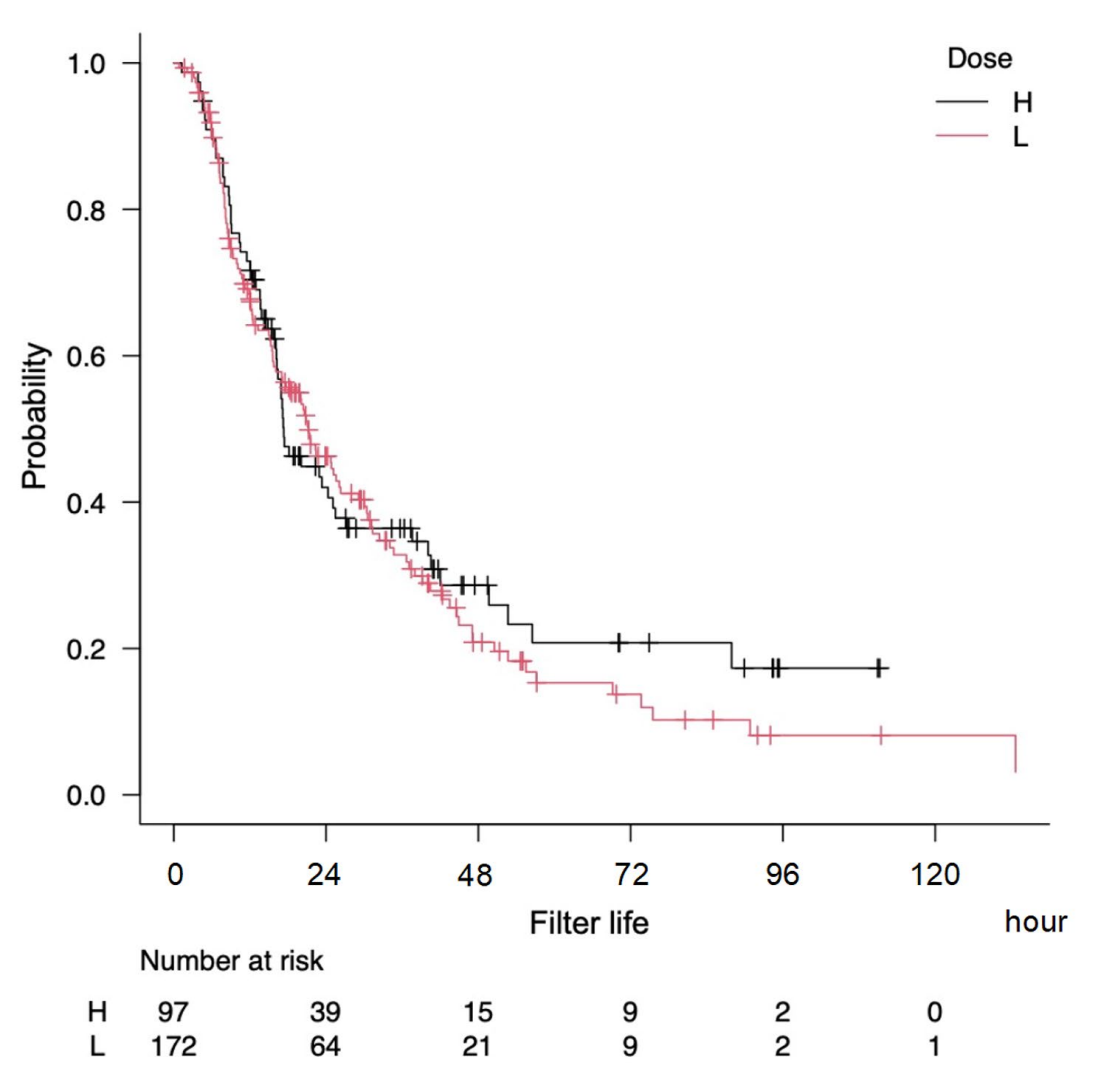
The probability of filter survival by the nafamostat mesylate dosage plotted in the Kaplan-Meier curve. Adjusted for bleeding risk and haemofiltration. Group H received nafamostat mesylate at the dose of ≥ 20 mg/hr. Group L received nafamostat mesylate at the dose of < 20 mg/hr

The median filter life, regardless of censoring, was 18.3 h (IQR, 9.3 to 36.7; mean, 26.4 h, SD, 23.4). The filter survival analysis, which categorised patients into high and low-dose groups, showed no significant association between the filter life and nafamostat mesylate dosage. After adjusting for bleeding risk and the addition of haemofiltration to haemodialysis, the hazard ratio was 1.12; 95 CI 0.74–1.69, *p* = 0.60), as shown in Fig. 1.

As for the secondary outcomes (Table 4), the mean ICU length of stay were 14 days (SD, 21.1). Of note, 68 / 182 (37.4% of those who had not been on chronic dialysis at ICU admission) patients were dialysis dependent at hospital discharge. From the generalised linear regression analyses, adjusting for potential confounders (eTable 2), no association between nafamostat dosage and ICU length of stay, hospital length of stay, duration of mechanical ventilation, or CRP was observed. However, decreasing trends were observed in ICU mortality, in-hospital mortality, and the risk of dialysis dependence at discharge as the nafamostat dose increased. Also, a decreasing trend in the amount of blood transfusion within 48 h after the start of CKRT was observed as nafamostat mesylate increased.

**Table 4:** Clinical outcomes and the association between nafamostat dose and outcomes. The association with each outcome was assessed using distinct generalised linear models, each adjusted for confounding factors appropriate to the respective outcome, as detailed in eTable 2

Outcome	Overall (*n* = 269)	Adjusted OR	β	95%CI	*p* value
ICU mortality	61 (22.7%)	0.95		(0.90, 0.99)	0.04
Hospital mortality	94 (34.9%)	0.95		(0.91, 0.99)	0.03
ICU length of stay, day	14 ± 21.1		0.04	(-0.35, 0.44)	0.83
Hospital length of stay, day	66 ± 91.6		0.17	(-1.55, 1.89)	0.85
Mechanical ventilation days, day	8 ± 17.9		0.13	(-0.11, 0.37)	0.30
ΔC-reactive protein, mg/dL	1.49 ± 9.21		-0.03	(-0.15, 0.09)	0.67
Red blood cell over 48 h, ml	200.5 ± 377.8		-13.7	(-19.9, -7.5)	< 0.001
Fresh frozen plasma over 48 h, ml	241.1 ± 667.1		-22.9	(-34.1, -11.7)	< 0.001
Platelet over 48 h, ml	64.0 ± 162.5		-4.1	(-6.9, -1.2)	0.01
Dialysis dependence at discharge*	68 / 182 (37.4%)	0.95		(0.90, 1.00)	0.05

Values are presented as medians with interquartile range or means with standard deviations. OR denotes odds ratio; CI, confidence interval. *Among patients who had not been on chronic haemodialysis

## Discussion

### Summary of the key findings

In this observational study, nafamostat mesylate was empirically used within the range of 5–30 mg/hr during CKRT as usual practice. There was no significant association between filter life and doses of nafamostat mesylate.

### Context with prior literature

Despite its widespread use for CKRT, particularly in Japan, there are currently no evidence-based guidelines for the indication of nafamostat mesylate. This absence of data-driven guidelines has led to nafamostat mesylate being used based on empirical evidence, often in scenarios with a high risk of bleeding, but without the backing of robust clinical data. The current study highlights the urgent need for comprehensive research to establish evidence-based practices and guidelines for the use of nafamostat mesylate for CKRT.

No previous studies have investigated the dose responsive anticoagulant effect of nafamostat mesylate on filter life when used for CKRT. It is important to note that the administration of nafamostat mesylate currently varies with dosages based on the individual experiences of different facilities, physicians, or technicians. Therefore, we explored the association between nafamostat prescription with experience based sterategy and filter life. Our findings showed no significant relationship between filter life and the doses of nafamostat mesylate.

The recommended dosage of nafamostat mesylate indicated in the brochure ranges from 20 to 50 mg/h. However, the dosage pertains to intermittent dialysis [12], and the optimal dosage for CKRT in critically ill patients remains undetermined [13]. The literature reports a range of nafamostat dosing. Two previous randomised clinical trials (RCTs) started nafamostat mesylate at 20 mg/h, adjusting between 10 mg/h and 30 mg/h based on individual patient’s condition and reported that nafamostat mesylate prolongs filter life effectively compared to the absence of anticoagulant [4, 9, 14]. Conversely, some observational studies reported that the initial dose of 10 mg/h. The reasons for the reduced dose, compared to the recommended dose, might be empirical considerations and the absence of safety data on for CKRT. In other observational studies of patients with cerebral haemorrhage, nafamostat mesylate was administered at a rate of 35 mg/h [15]. Another observational study comparing nafamostat mesylate with citrate administrate nafamostat mesylate with a regimen of 1 mg/kg as a bolus, followed by a maintenance dose of 1 mg/kg/hr, with monitoring via activated clotting time [16].

The mean filter life in this study was 26.4 h. This aligns with previous studies that reported filter lives ranging from 22 to 26.6 h when using nafamostat [17], suggesting that the current study findings could be could be applicable the clinical practice when using nafamostat mesylate in other settings. However, the filter life time around 24 h might not be comparable to ones when other anticoagulants are used (RICH tial) [18]. In the previous clinical trial, regional citrate anticoagulation provided the mean filter life of 44.9 h, while that of 33.3 h when unfractionated heparin was used. Of note, the blood flow rate in the current study settings was low, thus the filter life might have been potentially shortened, while the impact of blood flow rate on filter life has not been definitively concluded [19].

According to a recent systematic review [14], efficacy of nafamostat mesylate has been examined in randomised clinical trials only in comparison with no anticoagulation strategy. Previous observational studies have compared nafamostat mesylate with various anticoagulants, including no anticoagulation, heparin sodium, and citrate, particularly focusing on outcomes like filter life and bleeding events [9, 10, 16, 20, 21]. However, the results from these observational studies have been varied. Consequently, there is currently insufficient evidence to conclusively determine whether the observed filter life in using nafamostat mesylate is comparable to other anticoagulants.

Possible reasons for the observed decreasing trend in ICU and hospital mortalities with increased nafamostat mesylate dose are unclear. However, they may include that nafamostat may have beneficial effects beyond the anticoagulant properties. Nafamostat mesylate is also known for its antifibrinolytic [22] and anti-platelet [23] effects as partly explored in COVID-19. This could imply that nafamostat mesylate may play a role in decreasing of the underlying pathologies in critical illness. Furthermore, the decreasing trend of the required transfusion volume might also indicate a favourable safety profile for the nafamostat mesylate, especially if the higher doses do not lead to increased bleeding risks but instead associated with improved clinical outcomes.

However, the mechanisms behind the observed decrease in mortality and transfusion with increased nafamostat mesylate dosage remain unclear. Therefore, it is essential to investigate whether nafamostat mesylate has causative effects on patient outcomes. In this regard, the current findings needs to be further validated in a randomised trial comparing different doses of nafamostat mesylate for CKRT. The findings will inform further investigation on the optimal dose of nafamostat mesylate to provide adequate filter life with established safety profile. If the optimal dosage of nafamostat mesylate is known, further comparative studies with other drugs (heparin sodium, citrate, etc.) would be possible.

### Limitations

This study has several limitations. First, while we adjusted for possible confounders in patient backgrounds, filter types, severity, and filtration volumes, there might be unmeasured confounders that had not been sufficiently adjusted for. In particular, the observed decreased mortalities might be driven by confounding by the indication; that is, clinicians prescribed higher dose of nafamostat mesylate because the patient had been less sick and at lower risk of bleeding. Second, it is also pertinent to note that as an observational study, the findings should be considered exploratory given the possibility of bias due to unmeasured confounders. However, our results may inform the design of future RCTs controlling patient background characteristics and CKRT settings to find the optimal dose of nafamostat mesylate. Third, adjusting doses after initiating CKRT may differ due to the lack of a standardised protocol for achieving an optimal anticoagulation level to maintain circuit patency. Fourth, the causes of deterioration of kidney function or the indications to start CKRT were not specified in medical records; thus, we were unable to systematically collect the information to precisely describe them. Instead, we collected and analysed laboratory data that could reflect the patients’ conditions at the time of initiating CKRT to provide insights into their conditions. Fifth, the study was conducted where relatively low-intensity CHD was delivered. This might have influenced the clearance of nafamostat mesylate, thereby limiting the generalisability of the findings in different clinical settings. Finally, being conducted in two ICUs, the sample size of the study was relatively small, which might limit the power to detect a dose-response relationship in the study population.

## Conclusions

We observed no clear dose-response relationship between the nafamostat mesylate dose ranging from 5 to 30 mg/h during CKRT and the filter life in critically ill patients. Increasing the dose of nafamostat mesylate in this range appeared to be safe, which warrants randomised clinical trials to find the optimal dose in the future.

### Electronic supplementary material

Below is the link to the electronic supplementary material.

Supplementary Material 1

## Data Availability

The datasets used and/or analysed during the current study are available from the corresponding author on reasonable request.

## Abbreviations

CKRT: Continuous kidney replacement therapy
ICU: Intensive care unit
AKI: Acute kidney injury
WBC: White blood cell
CRP: C-reaction protein
CI: Confidence interval
NYHA: New York Heart Association
APACHE: Acute physiology and chronic health evaluation
APTT: Activated partial thromboplastin time
PT-INR: Prothrombin time-international normalized ratio
AN69ST: Polyethylenimine-coated polyacrylonitrile
CTA: Cellulose triacetate
PS: Polysulfone
SD: Standard deviation
IQR: Interquartile range

## References

[1] Iwagami M (2015). Current state of continuous renal replacement therapy for acute kidney injury in Japanese intensive care units in 2011: analysis of a national administrative database. Nephrol Dial Transpl.

[2] Uchino S (2003). Continuous is not continuous: the incidence and impact of circuit down-time on uraemic control during continuous veno-venous haemofiltration. Intensive Care Med.

[3] Kidney Disease: Improving Global Outcomes Acute Kidney Injury Work Group (2012). KDIGO Clinical Practice Guideline for Acute kidney Injury. Kidney Int Suppl (2011).

[4] Choi JY (2015). Nafamostat Mesilate as an anticoagulant during continuous renal replacement therapy in patients with high bleeding risk: a Randomized Clinical Trial. Med (Baltim).

[5] Shinoda T (2010). Anticoagulation in acute blood purification for acute renal failure in critical care. Contrib Nephrol.

[6] Hanafusa N (2015). Application of continuous renal replacement therapy: what should we consider based on existing evidence?. Blood Purif.

[7] *National Center for Biotechnology Information. PubChem Compound Summary for CID 4413, Nafamostat*

[8] Lin Y (2022). Efficacy and safety of nafamostat mesilate anticoagulation in blood purification treatment of critically ill patients: a systematic review and meta-analysis. Ren Fail.

[9] Lee YK (2014). Ability of nafamostat mesilate to prolong filter patency during continuous renal replacement therapy in patients at high risk of bleeding: a randomized controlled study. PLoS ONE.

[10] Makino S (2016). Comparison of nafamostat mesilate and unfractionated heparin as anticoagulants during continuous renal replacement therapy. Int J Artif Organs.

[11] von Elm E (2007). Strengthening the reporting of Observational studies in Epidemiology (STROBE) statement: guidelines for reporting observational studies. BMJ.

[12] Koshikawa S (1987). Clinical evaluation of FUT-175 as a regional anti-coagulant in hemodialysis: multi-center cooperative study. J Jpn Soc Dialysis Ther.

[13] Kameda S (2023). Unfractionated heparin versus nafamostat mesylate for anticoagulation during continuous kidney replacement therapy: an observational study. BMC Nephrol.

[14] Tsujimoto H (2020). Pharmacological interventions for preventing clotting of extracorporeal circuits during continuous renal replacement therapy. Cochrane Database Syst Rev.

[15] Yang JW (2009). Superior outcome of nafamostat mesilate as an anticoagulant in patients undergoing maintenance hemodialysis with intracerebral hemorrhage. Ren Fail.

[16] Miyaji MJ, et al. Comparison of nafamostat mesilate to citrate anticoagulation in pediatric continuous kidney replacement therapy. Pediatr Nephrol; 2022. 10.1007/s00467-022-05502-8 35348901

[17] Zhang W (2021). Continuous renal replacement therapy without anticoagulation in critically ill patients at high risk of bleeding: a systematic review and meta-analysis. Semin Dial.

[18] Zarbock A (2020). Effect of Regional Citrate Anticoagulation vs systemic heparin anticoagulation during continuous kidney replacement therapy on Dialysis Filter Life Span and Mortality among critically ill patients with acute kidney Injury: a Randomized Clinical Trial. JAMA.

[19] Tsujimoto Y (2021). Non-pharmacological interventions for preventing clotting of extracorporeal circuits during continuous renal replacement therapy. Cochrane Database Syst Rev.

[20] Baek NN (2012). The role of nafamostat mesylate in continuous renal replacement therapy among patients at high risk of bleeding. Ren Fail.

[21] Hwang SD (2013). Nafamostat mesilate for anticoagulation in continuous renal replacement therapy. Int J Artif Organs.

[22] Asakura H (2021). COVID-19-associated coagulopathy and disseminated intravascular coagulation. Int J Hematol.

[23] McFadyen JD (2020). The emerging threat of (Micro)thrombosis in COVID-19 and its therapeutic implications. Circ Res.

